# Manganese-Enhanced MRI: Biological Applications in Neuroscience

**DOI:** 10.3389/fneur.2015.00161

**Published:** 2015-07-10

**Authors:** Jackeline Moraes Malheiros, Fernando Fernandes Paiva, Beatriz Monteiro Longo, Clement Hamani, Luciene Covolan

**Affiliations:** ^1^Department of Physiology, Universidade Federal de São Paulo – UNIFESP, São Paulo, Brazil; ^2^Centro de Imagens e Espectroscopia In vivo por Ressonância Magnética, Institute of Physics of São Carlos, Universidade de São Paulo, São Carlos, Brazil; ^3^Research Imaging Centre, Centre for Addiction and Mental Health, Toronto, ON, Canada; ^4^Centre for Addiction and Mental Health, Campbell Family Mental Health Research Institute, Toronto, ON, Canada

**Keywords:** manganese, tracing method, epilepsy, nociception, anatomy, MRI

## Abstract

Magnetic resonance imaging (MRI) is an excellent non-invasive tool to investigate biological systems. The administration of the paramagnetic divalent ion manganese (Mn^2+^) enhances MRI contrast *in vivo*. Due to similarities between Mn^2+^ and calcium (Ca^2+^), the premise of manganese-enhanced MRI (MEMRI) is that the former may enter neurons and other excitable cells through voltage-gated Ca^2+^ channels. As such, MEMRI has been used to trace neuronal pathways, define morphological boundaries, and study connectivity in morphological and functional imaging studies. In this article, we provide a brief overview of MEMRI and discuss recently published data to illustrate the usefulness of this method, particularly in animal models.

## Introduction

Magnetic resonance imaging (MRI) is an excellent non-invasive tool for providing anatomical information of biological systems (1–6) due to its unique soft tissue contrast and relatively high-spatial resolution.

With a large variety of MRI applications being proposed, great effort has been made to develop contrast agents that may add physiological and/or molecular information to anatomical images (7). Along this line, the potential use of the paramagnetic manganese ion (Mn^2+^), which induces a strong reduction in both longitudinal (T_1_) and transversal (T_2_) relaxation times, has been investigated (8). As Mn^2+^ has a high-chemical similarity with calcium (Ca^2+^), it may enter neurons and other excitable cells through voltage-gated calcium channels and the Na^+^/Ca^2+^ exchanger (9).

Over the last decade, Mn^2+^ has been used as a contrast agent in various manganese-enhanced MRI (MEMRI) applications. These may be grouped in three major classes: neuronal tract tracing (10–14), morphological (15–18), and functional imaging (19–23). Typically, during neuronal tract-tracing studies manganese is directly injected into a specific brain region (24–29). In other classes of applications, this ion is administered either systemically into the bloodstream (30–39) or directly into the cerebrospinal fluid (CSF) (40, 41).

Neuronal tract-tracing explores the transport of Mn^2+^ across synapses. In contrast, morphological and functional studies using MEMRI are dependent on local neuronal cell density, the permeability of the blood–brain barrier, and neuronal activation (42). In Mn^2+^-based functional MRI (fMRI), tissue contrast may be correlated with activity-dependent ion accumulation in excitable cells (43). As such, the contrast in MEMRI is more directly related to neural activity then fMRI blood oxygenation level dependent (BOLD) (44, 45). Another advantage is that Mn^2+^ uptake after systemic injections takes place over an extended period of time in awake and freely moving animals (46). As a result, only the MRI acquisition needs to be performed under anesthesia. This is another advantage of MEMRI over BOLD fMRI, which requires both stimuli and acquisition to be performed under sedation.

A major drawback of the use of Mn^2+^ is the toxic side effects observed at high concentrations (47–51). This is of concern as high-Mn^2+^ tissue levels are often required to enhance the contrast between structures (52–54). In fact, toxicity is one of the main limitations for the full development of Mn^2+^ as an MRI contrast agent for humans. Even in animal studies, there needs to be a compromise between avoiding toxicity and delivering adequate doses of manganese. The ultimate goal is to reduce systemic side effects while guaranteeing animal well-being and maximizing contrast and imaging quality (8, 17, 55).

Several methodological developments have been recently proposed to improve MEMRI as a technique to study functional neural circuits and *in vivo* brain anatomy. In the present work, we provide a brief overview of MEMRI and illustrate the potential applications of this method in small animal models.

## Manganese-Enhanced MRI

### Historical perspective

The first use of Mn^2+^ in nuclear magnetic resonance (NMR) coincides with the early days of this technique (56). Together with other ions, Mn^2+^ was employed in tests to measure the exchange rate of bulk water molecules with those in the first coordination sphere of paramagnetic ions (56). These findings played an important role in our understanding and optimization of water-exchange effects, a crucial step in the development of efficient T_1_-shortening MRI contrast agents (57–59). Later, Mn^2+^ was also used in experiments that enabled quantitative structural information to be obtained from biological molecules, which led to the development of techniques to determine protein structure using NMR (60).

Mn^2+^ has also been present since the earliest stages of MRI. Lauterbur (61) has used MnSO_4_ to change the longitudinal relaxation time of water and prove that relaxation times could affect signal intensity. This was an important step to demonstrate the feasibility of MRI, since, at that time, the technique was believed to be limited due to the small variations of water density in biological tissues (62). Mn^2+^ can then be considered as the first reported MRI contrast agent. Since then, it has contributed to our understanding of relaxation effects in biological systems (63). These are still considered to be helpful in establishing strategies to alter MRI contrast with exogenous agents and are extremely useful, not only in clinical practice but also in preclinical models (64, 65).

### Dosage and toxicity

The ion Mn^2+^ is essential for a normal development and cellular function. Disruptions in manganese homeostasis in humans are associated with neurological disorders, skin lesions, bone diseases, and among others (66–68). Chronic exposure to this heavy metal leads to manganism, a progressive neurodegenerative condition that resembles Parkinson’s disease (47, 50, 69, 70). An acute overexposure to Mn^2+^, which happens when a high-systemic dose of contrast agents is administered to patients, may result in cardiac toxicity, hepatic failure, and even death (48, 49, 71).

As the MEMRI contrast is proportional to the accumulation of tissue Mn^2+^ (52–54), the successful application of this technique depends on the delivery of appropriate ionic doses to the regions of interest. The most common way for delivering Mn^2+^ is through the injection of MnCl_2_ solutions (8). Depending on the application, MnCl_2_ can be delivered directly into the brain. This minimizes toxicity, since the exposure to lower doses of Mn^2+^ is restricted to the injection site and adjacent regions. Though focal toxicity may still occur (72), this approach has been successfully used in several studies of neuronal tract tracing (24–29).

For systemic injections targeting the brain, MnCl_2_ can be injected intravenously, intraperitoneally, or subcutaneously. So far, all have been widely used, as there is no strong evidence suggesting that one route is better or causes more toxicity than the others (30, 31, 33–39). One of the major drawbacks of using systemic injections is that, prior to reaching the brain manganese reaches the liver, heart, and kidneys. This increases the risk of acute toxic effects, including cardiac, renal, and liver failure.

In the intact brain [i.e., without blood–brain barrier (BBB) breakdown], the time-course and distribution of MnCl_2_ varies across brain regions (34, 73). Under these circumstances, contrast enhancement seems to reach its equilibrium 24 h following administration. As this is particularly slow for brain activation studies, one strategy is to disrupt the BBB to accelerate uptake (19, 43, 46). An alternative to avoid BBB disruption (40, 41) is to administer MnCl_2_ directly into the CSF. In this case, Mn^2+^ is uniformly supplied to the whole brain in a reasonable timescale for a variety of chronic functional activation studies.

The use of systemic fractionated injections (limited to small daily doses) was proposed as an alternative for delivering high doses of Mn^2+^ with fewer side effects in preclinical models (52, 53). A similar increase in contrast delivery with low toxicity has been observed with the use of subcutaneous mini-osmotic pumps (74). It is important to mention, however, that studies using these techniques were designed to demonstrate alternative ways of improving MRI contrast enhancement. Every attempt to use similar protocols should take into account reported changes in behavioral, neurochemical, electrophysiological, and histological signs of toxicity, especially when considering long-term effects (75–78).

### Routes of administration

In general, the route of delivery (i.e., systemic or intracerebral) is chosen based on the application. After the systemic administration, most Mn^2+^ likely reaches the brain through the blood–CSF barrier (79), enhancing the visualization of the cerebral cytoarchitecture and demarcating active brain regions. The focal cerebral administration enables mapping of neuronal tracts in the living brain, where Mn^2+^ is stored and transported along axonal tracts (75). As already mentioned, MEMRI applications can be grouped into three major classes: morphological (15–18), neuronal tract tracing (6, 10–14), and functional imaging (19–23).

In contrast to gadolinium-based agents that are typically intravascular and remain in the cerebral vasculature, MEMRI contrast achieved after the systemic administration of Mn^2+^ comes from the brain parenchyma itself. Mn^2+^ may enter the brain basically through three different routes are as follows: (i) from the bloodstream via a fast transport system in the choroid plexus. Through this route, Mn^2+^ gets very rapidly into the CSF and brain (80, 81); (ii) from the nasal space through the olfactory nerve via olfactory epithelium (25, 82, 83); (iii) from the bloodstream across the BBB at cerebral capillaries (84–87). In the intact brain, MEMRI signal enhancement following Mn^2+^ administration begins in the ventricles and periventricular regions prior to reaching more distant areas of brain parenchyma (34, 80, 88).

Once in the brain, manganese may be transported along axons (89) or across synapses (26). The time-course and distribution of MnCl_2_ varies across brain regions (34, 73). Those with an initial poor access to manganese may be supplied over time by axonal transport from areas with a strong initial uptake (88). Contrast enhancement seems to reach its equilibrium 24 h following administration. Thereafter, manganese has an extremely slow clearance rate that can take up to 300 days, with a half-life of 51–74 days in different brain regions, as shown by autoradiography (90). MRI-based studies showed a reduced Mn^2+^ half-life of 5–12 days, but not of the same magnitude (54, 91, 92). Since the regional signal enhancement following manganese administration is proportional to the propensity of each brain region to uptake this metal, MEMRI is a powerful tool for visualizing brain architecture.

### Manganese entrance into excitable cells

Overall, Mn^2+^ presents a high-chemical similarity with calcium (Ca^2+^), being handled in an analogous manner by many biological systems (93). This means that the Mn^2+^ can enter neurons and other excitable cells through calcium pathways, such as voltage-gated calcium channels and the Na^+^/Ca^2+^ exchanger (9, 86). In addition, Mn^2+^ can bind to intracellular proteins and nucleic acids. Once in the cell, Mn^2+^ accumulates in the endoplasmic reticulum (25, 26), being subsequently packaged into vesicles and transported anterogradely in axonal tracts. Upon reaching the presynaptic membrane (27, 89), it is finally released and taken up by the next neuron (25, 27). This property, along with the fact that Mn^2+^ is MRI-detectable, has contributed to its labeling as an *in vivo trans*-synaptic tracer.

Prior to MEMRI, tract-tracing studies employed invasive techniques (94, 95), requiring tracers to be injected and animals sacrificed in order for these agents to be visualized. A major limitation of this methodology is that longitudinal studies cannot be carried out in the same animals. As MEMRI can be conducted multiple times, it has contributed to the *in vivo* temporal assessment of connectivity and integrity of neuronal tracts in several animal models (i.e., from small rodents to non-human primates) (13, 26, 28, 96).

The ability of manganese to be taken up via voltage-gated Ca^2+^ channels has not only been explored for non-invasive tract tracing but also to functionally assess the rate of neuronal transport. This latter plays a crucial role in the normal functioning of neurons. In fact, perturbations in axonal transport and its machinery have been associated with disease states, such as Alzheimer’s disease, diabetes, as well as with normal aging (97–99). In contrast to Mn^2+^, large tracer molecules may not accurately represent the axonal transport in *in vivo* systems.

### Activity-induced manganese MRI

The main concept underlying the use of MEMRI for the assessment of neuronal activity is the fact that activated brain regions have elevated Ca^2+^ influx through Ca^2+^ channels. As mentioned before, in the presence of extracellular Mn^2+^ active regions will have greater Mn^2+^ influx, since manganese competes with Ca^2+^ to enter the cells. Thus, the accumulation of Mn^2+^ is directly related to brain activation and may provide information about brain function. This approach, which has been named activity-induced manganese MRI (46), led to the development of a Mn^2+^-based fMRI technique. It differs from traditional methods, because it does not take into account information on hemodynamic fluctuations and deoxy-hemoglobin concentration. Hence, the activity-induced manganese-dependent contrast (AIM) MRI produces maps with better spatial localization than those produced by conventional fMRI (19).

A particular concern related to AIM MRI experiments is that the Mn^2+^ cannot efficiently penetrate the BBB. The CSF route is particularly slow for this purpose (87, 100) and the amount of Mn^2+^ entering the brain is minimal compared to cases where the BBB is disrupted. As a result, several AIM MRI studies have been performed in conjunction with BBB disruption. On the other hand, some studies showing activation of the auditory (22, 23) and visual pathways (30, 101) following auditory and visual stimulation, respectively, were performed in mice without BBB disruption.

An interesting aspect of AIM MRI is that, after BBB disruption and upon brain stimulation, Mn^2+^ accumulates in active regions at a short time scale. Once accumulated, Mn^2+^ does not leave these regions for several hours. This allows Mn^2+^ to be delivered outside the scanner, while the animal is being freely moving or carrying out behavioral tasks. When compared with conventional fMRI protocols, this represents a new horizon in terms of functional evaluation. One of its disadvantages, however, is the intrinsic temporal resolution of the technique, which prevents the assessment of rapid changes in activity, particularly tissue deactivation (102). Besides providing valuable information to answer physiological questions, AIM MRI was proven to be an important tool for the study of spatial BOLD signal changes in the cortex (19, 45, 103, 104). This is particularly important because BOLD is the MRI-based “gold standard” method for measuring brain activity in humans and several methodological questions still remain to be addressed.

## MEMRI: Recent Applications in Experimental Animal Models

Over the last years, MEMRI has been extensively used in neurosciences. Studies using this technique have addressed neurophysiological and neuroanatomical problems in animal models of nociception (105, 106), neurodegeneration (35, 36, 99, 107–111), and psychiatric disorders (112).

### Activity-dependent signaling

In animals, MEMRI has been used to determine high versus low activation of brain areas after specific stimuli or in models of brain disease. One example is the sequence of activation of the hypothalamic paraventricular nucleus, supraoptic nucleus, and preoptic area, which are thought to be involved in central osmotic regulation after intracarotid injection of hypertonic NaCl (113). In another study, mice exposed to an odorant showed localized T_1_ MRI signal enhancements in the olfactory epithelium and bulb (25). MEMRI has also been shown to be effective for mapping the mouse auditory brainstem (22). Chronic tinnitus (the perception of sounds in the absence of acoustic stimulation) in rats was associated with elevated focal activity in the auditory brainstem (114). On the other hand, a reduction in Mn^2+^ uptake was demonstrated in the rodent visual cortex in depression-like states (sickness behavior) induced by interferon-α (IFN-α), which was related to altered local functionality (112).

### Epilepsy

**Figure 1:**
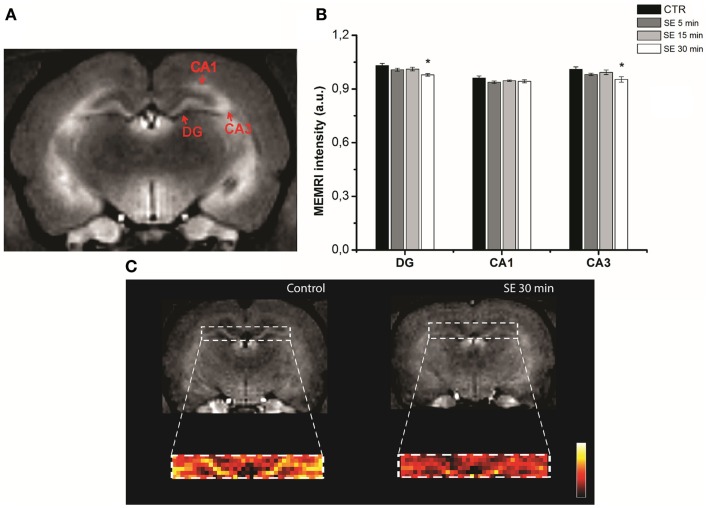
**Hippocampal MEMRI in pilocarpine injected animals, at different time points after status epilepticus (SE): 5 (SE 5 min), 15 (SE 15 min), and 30 min (SE 30 min)**. T_1_-weighted MEMRI images **(A,C)** and MEMRI data **(B)**. Regions of interest (ROIs) drawn in hippocampal sub regions, including the DG (dentate gyrus), CA1 and CA3 (Cornu Ammonis), are represented in **(A)**. The DG was enlarged and converted from gray into a colored scale in **(C)** to show differences between non-epileptic controls and the SE 30 group (*P < 0.01). Reproduced with permission from Malheiros et al. (35).

At first sight, these results may suggest a positive correlation between MEMRI enhancement and cell activation. However, other factors, such as tissue edema, neurodegeneration, and cell density (8), may also determine signal changes, as shown in animal models of epilepsy. Several rodents and non-human primate models have been used to study cellular mechanisms that underlie temporal lobe epilepsy (TLE), including those following pilocarpine, kainic acid (115–121), and pentylenetetrazol injections (122). In these models, status epilepticus (SE) represents an acute phase, after which the animals enter the silent period that ends with the occurrence of spontaneous recurrent seizures (chronic phase). The temporal sequence and the neuropathological alterations that characterize these chronic models resemble those observed in human TLE. In rodents, the acute phase of the kainic acid model is characterized by a poorly defined MEMRI signal in areas with high-cellular activity (i.e., hippocampus) (107, 108). A possible explanation for this finding is that the MEMRI signal may have been obscured by cell damage that occurs at this early phase, especially when SE lasts more than 30 min. Similar results have been shown during the acute phase of the pilocarpine model (35, 109), even when SE lasted only from 5 to 30 min (Figure 1). A proposed mechanism to explain this finding is that reductions in MEMRI signal could be related to hippocampal cell edema rather than apoptotic cell death (35). Both edema and cell death have to be taken into account when one is planning to map active or inactive brain areas with MEMRI.

As mentioned above, both the kainic acid and pilocarpine models exhibit spontaneous recurrent seizures in the chronic phase, which, as described in humans, are accompanied by hippocampal sclerosis and mossy fiber sprouting (MFS) (115, 116, 123, 124). MRI has been largely used to study the chronic phase of TLE, since it allows a non-invasive longitudinal follow up using different approaches. These include anatomical imaging for evaluating hippocampal and amygdala volumetric changes (110, 125–128) and relaxometry for estimating relaxation times changes in different brain areas (i.e., hippocampus, amygdala, piriform cortex, and/or thalamus) (127, 129–131). Longitudinal studies may also be used to evaluate changes in spectroscopy so that biochemical changes may be characterized. As an example, the hippocampi of lithium–pilocarpine-treated rats have reduced *N*-acetylaspartate (NAA) and choline (Cho) peaks, as well as an increase in lactate compared to non-epileptic controls (131). Besides these MRI approaches, MEMRI is used as a molecular imaging technique (35, 36, 107–110). The focal and systemic administration of MnCl_2_ results in an increased hippocampal dentate gyrus MEMRI signal in kainic acid (108, 110) and pilocarpine-chronic epileptic rats (36). In these animals, signal changes correlates with aberrant MFS.

**Figure 2:**
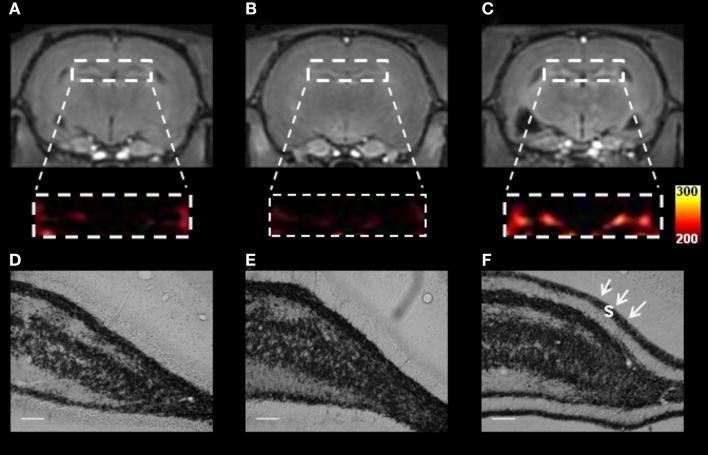
**T_1_-weighted MEMRI (A–C) and representative photomicrographs (D–F) of pilocarpine-chronic epileptic rats (Pilo-treated) with no mossy fiber sprouting (MFS, left panel), Controls (middle panel), and Pilo-treated MFS-positive (right panel) animals**. The dentate gyrus in MEMRI **(A–C)** was enlarged and converted from a gray into a colored scale. Pilo animals showed MFS in the supragranular layer (s, black arrowheads) and MEMRI hyperintensity in the DG. Similar abnormalities have not been detected in either epileptic animals with no MFS or controls. MRI data from the three groups were normalized to the muscle signal intensity to minimize possible signal differences. Scale bars = 50 μm. Reproduced with permission from Malheiros et al. (36).

The relationship between MFS and MEMRI hyperintensity in pilocapine animals can be observed in Figure 2. Chronic pilocarpine rats that show aberrant MFS also show MEMRI hyperintensity. These signal changes have not been observed in pilocarpine animals in which MFS was suppressed by cycloheximide, suggesting that (1) MEMRI is able to detect hippocampal changes during the course of epileptogenesis and (2) a relationship exist between manganese enhancement and spontaneous seizure outcome (132). From the above-mentioned results, we conclude that MEMRI is a useful tool to follow important aspects related to neuronal plasticity, including those related to aberrant MFS and spontaneous recurrent seizures. Unfortunately, however, MEMRI may not be useful to study-activated areas during the acute phase of these models, as injury-related edema interferes in the signal.

### Pain

In pain-related studies, MEMRI has been used to delineate functional connections between cortical and non-cortical areas; electrical stimulation of the left forepaw increased MEMRI signal in the contralateral anterior cingulate cortex, midcingulate cortex, retrosplenial cortex, ventral medial caudate-putamen, nucleus accumbens, and amygdala. Of those, signal changes in the retrosplenial cortex were attenuated by morphine injections (106). The efficacy of MEMRI to trace anatomical connections was indeed confirmed by Mn^2+^ transportation from the medial thalamus to the cingulate cortex and medial striatum, but not the motor cortex (106).

A recent study has shown reduced reactivity to thermal pain in the dorsal spinal cord following repeated amphetamine injections (133). The authors showed a temporal correlation between reduced pain sensitivity and increased MEMRI signals in the dorsal horn following repeated amphetamine administration. MEMRI has also been valuable in demonstrating the involvement of the hippocampus in the processing of pain during early development (105). As shown by different studies, noxious stimulation of newborn rats not only causes sex-specific long-term effects on the natural behavioral repertoire during adulthood (35, 134–136) but also dentate hippocampal cell activation.

In a rat model of pruritus, MEMRI has been used to investigate brain regions activated during itching. These were the parafascicular thalamic nucleus, superior/inferior colliculus, periaqueductal gray, cingulate cortex, amygdala, midbrain regions, lateral habenula, and hypothalamic areas (137). Gabapentin-treated itching rats decreased scratching behavior and had an attenuation of functional activity in the brain regions described above. Together, these results suggest that MEMRI hyperintensity is related to stimulus-induced activation of specific brain regions and that this techniques may be used as a strategy for understanding mechanisms of pain-related diseases.

### Axonal transport

Axonal transport is an essential physiological function. Its disruption severely interfere with neuronal viability and leads to distinct neurological disorders. As an example, axonal transport impairment occurs at the onset of optic neuritis in an experimental murine model of autoimmune encephalomyelitis (EAE). Using the MEMRI technique, it was demonstrated that Mn^2+^ accumulation and axonal transport were significantly decreased not only in these animals (138) but also in rTg4510 mice, which comprise a model of fronto-temporal dementia and parkinsonism (139). In a mouse model of Alzheimer’s, axonal transport rates were shown to be reduced as soon as amyloid-beta (Aβ) deposition begins. This reduction becomes even more pronounced after plaque formation (99). In this particular case, MEMRI showed that *in vivo* reduction in axonal transport can be detected prior to plaque formation.

### Mechanisms of pathological Mn^2+^ enhancement

Bearing in mind that Mn^2+^ enters neurons through Ca^2+^ channels and is transported along axonal transport systems, MEMRI has been used to trace the recovery of neuronal connectivity in experimental models of stroke (111). According to the authors, loss or dysfunction of neuronal connections, even outside the ischemic lesion, may explain the lasting impairment of function. Systemic Mn^2+^ injections in the acute phase of neonatal mild hypoxic–ischemia provide an enhanced MEMRI signal indicative of cortical gray matter lesion. This would be otherwise undetectable with conventional MRI techniques (140–142). In the late phase of the hypoxic–ischemia model, MEMRI signal was intense in the dorsolateral thalamus, hippocampus, and the remaining cortex of the injured hemisphere. This was co-localized with reactive astrocytes, dying neurons, and activated microglia on histological analysis. MEMRI enhancement in this study had higher correlation with activated microglia (suggesting inflammatory process) than with dying cells (143).

### Conclusions

Based on the above-mentioned studies, MEMRI may be considered as a powerful approach for *in vivo* studies to determine stimulus-dependent brain areas of activation, axonal transport, neuronal connectivity, and brain lesion in several experimental animal models. However, few challenges still have to be overcome so that researchers may take full advantage of all the benefits that this technique has to offer. Since dose-related toxicity is a major concern, there is a need to develop and further refine MRI pulse sequences in order to make them more sensitive to small changes in relaxation times. Also, it is important to develop better strategies to deliver the Mn^2+^ to the region of interest, reducing the risk of side effects after systemic MnCl_2_ injections. The combination of all of these aspects will likely allow MEMRI to be an even more powerful, versatile, and useful tool for modern neurosciences studies.

## Conflict of Interest Statement

The authors declare that the research was conducted in the absence of any commercial or financial relationships that could be construed as a potential conflict of interest.
